# Evaluating depressive symptoms in hypomanic and manic episodes using a structured diagnostic tool: validation of a new Mini International Neuropsychiatric Interview (M.I.N.I.) module for the DSM-5 'With Mixed Features’ specifier

**DOI:** 10.1186/2194-7511-1-21

**Published:** 2013-10-17

**Authors:** Thierry Hergueta, Emmanuelle Weiller

**Affiliations:** Département de Neurologie, IMMA Pavillon François Lhermitte, GHU Pitié-Salpêtrière, Charles Foix, 47, boulevard de l’hôpital, Paris, 75013 France; Corporate Medical Affairs, H. Lundbeck A/S, Ottiliavej 9, Valby, 2500 Denmark

**Keywords:** Bipolar disorder, Depressive symptoms, DSM-5, Mania, Mini International Neuropsychiatric Interview, Mixed episode, Mixed features, Structured diagnostic interview, Validation

## Abstract

**Background:**

The Diagnostic and Statistical Manual of Mental Disorders, fifth edition (DSM-5), includes a new 'With Mixed Features’ specifier for mood episodes. In (hypo-)manic episodes, the specifier is given if three or more depressive symptoms are present nearly every day during the episode. A new module of the Mini International Neuropsychiatric Interview (M.I.N.I.) has been developed as a patient-completed questionnaire to evaluate the DSM-5 specifier for (hypo-)manic episodes. The objective of this study was to validate this new module.

**Methods:**

In Phase I, patients with a manic episode in the past 6 months completed the module and were asked whether the wording was clear, understandable, relevant and specific. Based on their feedback, the module was refined and finalised. In Phase II, psychiatrists each invited five patients to complete the module. The psychiatrists completed record forms for these five patients, which included their diagnoses, made according to DSM-5 criteria during clinical interviewing. The module was validated by comparing depressive symptoms reported by the patients themselves using the M.I.N.I. module with those evaluated by their psychiatrist using DSM-5 criteria during clinical interviewing.

**Results and discussion:**

In Phase I, a few changes were made to the M.I.N.I. module based on feedback from 20 patients (60% of whom had mixed features). In Phase II, 23 psychiatrists completed record forms for 115 patients, 99 (86.1%) of whom completed the M.I.N.I. module. Agreement between psychiatrists' DSM-5 diagnoses and patients' M.I.N.I. responses was substantial (Cohen's kappa coefficient, 0.60). The overall sensitivity of the M.I.N.I. was 0.91 and its specificity was 0.70. Sensitivity ranged from 0.63 for psychomotor retardation to 0.90 for suicidal thoughts. Specificity ranged from 0.63 for diminished interest/pleasure to 0.90 for suicidal thoughts. The module's positive and negative predictive values were 0.72 and 0.90, respectively. In summary, the M.I.N.I. module demonstrated good concurrent validity with psychiatrists' evaluation of DSM-5 mixed features in manic patients, accurately detecting mixed features with limited risk of over-diagnosis. Due to its simplicity, the M.I.N.I. module could be incorporated into routine psychiatric evaluation of patients with manic episodes. It could also provide a valuable standardised tool for clinical and epidemiological research.

**Electronic supplementary material:**

The online version of this article (doi:10.1186/2194-7511-1-21) contains supplementary material, which is available to authorized users.

## Background

Mood disorders are commonly divided into three major categories: unipolar mood disorders (major depressive disorder, dysthymic disorder), bipolar mood disorders (bipolar I disorder, bipolar II disorder, cyclothymic disorder) and mood disorders with a known aetiology (substance-induced mood disorder, mood disorder due to a general medical condition) (American Psychiatric Association 2000). Depression and mania are often considered to be at opposite ends of the mood spectrum, but they can coexist in what are termed 'mixed states’. Bipolar disorder is a chronic disease characterised by periods of mania or hypomania (episodes of elevated moods, extreme irritability, decreased sleep and increased energy), depression (overwhelming feelings of sadness, anhedonia, suicidal thoughts) or a combination of both (mixed states). The prevalence of bipolar disorder is estimated to be approximately 1% to 2% (Fagiolini et al. 2013).

In the American Psychiatric Association's Diagnostic and Statistical Manual of Mental Disorders, fourth edition-text revision (DSM-IV-TR), the diagnostic criteria for a 'mixed episode’ include a requirement that the patient meets the criteria for both a manic episode and a major depressive episode (except for duration) nearly every day during at least a 1-week period (American Psychiatric Association 2000). When reviewing the latest research evidence for the Diagnostic and Statistical Manual of Mental Disorders, fifth edition (DSM-5), published in May 2013, it was recognised that individuals rarely meet the full criteria for both a manic episode and major depressive episode at the same time, meaning that patients with mixed manic and depressive symptoms may not necessarily be accurately diagnosed and receive the most appropriate treatment (American Psychiatric Association 2013a). Therefore, the DSM-IV-TR criteria for a mixed episode have been removed and replaced in DSM-5 with a new 'With Mixed Features’ specifier for mood episodes, which can be applied to episodes of hypomania, mania or major depression (American Psychiatric Association 2013a). For hypomanic or manic episodes, the specifier is given if at least three of six defined depressive symptoms are present nearly every day during the most recent week of a manic episode or the most recent 4 days of a hypomanic episode. These depressive symptoms are 'prominent dysphoria or depressed mood’ , 'diminished interest or pleasure in activities’ , 'psychomotor retardation’ , 'fatigue or loss of energy’ , 'feelings of worthlessness or excessive/inappropriate guilt'’ and 'recurrent thoughts of death/suicide’ (American Psychiatric Association 2013a). Since manic episodes with depressive symptoms are generally more severe and have a poorer prognosis than pure manic episodes (González-Pinto et al. 2007, 2011; Goldberg and McElroy 2007; Valentí et al. 2011), and because these mixed states are common (affecting approximately one third of patients with bipolar disorder (González-Pinto et al. 2007, 2011)), it is hoped that this new specifier will enable clinicians to diagnose patients suffering from concurrent symptoms of mania/hypomania and depression more accurately. This will allow clinicians to better tailor treatment to their patients' particular needs (American Psychiatric Association 2013a). Furthermore, since mixed states are not easily identified by clinicians, it is crucial to systematically search for coexisting depression when a patient has a manic episode in order to ensure a correct diagnosis.

Structured diagnostic interviews have become an integral part of psychiatric medicine, not only being considered the 'diagnostic gold standard’ in psychiatric research but also increasingly being used to help ensure diagnostic precision in clinical practice (Nordgaard et al. 2012). Whereas information collected from open clinical interviews may vary depending on how a particular question is asked or framed, structured diagnostic interviews comprise questions that are precise and carefully linked to diagnostic criteria, minimising the risk of 'grey area’ and associated variability. Structured diagnostic interviews include the Mini International Neuropsychiatric Interview (M.I.N.I.), which was developed as a simple tool to assist clinicians to conduct psychiatric diagnoses according to the DSM-IV and International Statistical Classification of Diseases and Related Health Problems tenth revision (ICD-10) criteria (Lecrubier et al. 1997; Sheehan et al. 1998). The M.I.N.I has become a reference worldwide and been translated into 65 languages. Recently, a new module has been developed to evaluate the DSM-5 specifier 'With Mixed Features’ for hypomanic and manic episodes in a version that can be completed by patients themselves, since self-assessment in acute mania has been shown to be both feasible and valid (Hantouche et al. 2001).

Presented here are the results of a study that was conducted in order to validate the new M.I.N.I. module in patients with manic episodes, by assessing the degree of agreement between patients' M.I.N.I. module responses and the diagnoses made by psychiatrists using DSM-5 criteria during clinical interviewing.

## Methods

This was a prospective, real-world study, involving psychiatrists and patients with bipolar mania, which was conducted in the UK in November 2012. The study was divided into two phases. Phase I involved verification of bipolar patients' acceptance and comprehension of the questions comprising the M.I.N.I. module. Phase II involved assessment of the degree of agreement between patients' responses using the M.I.N.I. module versus DSM-5 criteria as evaluated by psychiatrists.

### Phase I: verification of bipolar patients' acceptance and comprehension of the questions

Twenty patients with bipolar disorder who had experienced a manic episode within the previous 6 months completed the initial version of the M.I.N.I. module. Patients were recruited using a quota system to achieve an even mix of subjects currently experiencing two or less or at least three depressive symptoms. The patients were asked whether the wording of the introductory sentence and each individual question of the M.I.N.I. module was clear, understandable, relevant and specific. Based on the patients' feedback, the wording was refined on a question-by-question basis during a consensus meeting of the study team, which was composed of the authors and methodologists. This refinement was based on a precise synthesis of the patients' answers (i.e. percentages of responses), together with their feelings about the module. The adapted module was then finalised before being used in Phase II.

### Phase II: assessment of the agreement between patients' responses versus DSM-5 criteria as evaluated by psychiatrists

#### Psychiatrist and patient selection

In order to be included in the study, physicians were required to be psychiatrists, qualified for 3 to 30 years, with a patient caseload comprising a minimum of 15 bipolar I patients per month. They were also required to be actively managing and treating patients with bipolar I disorder (i.e. to be the patients' 'decision maker’), to agree that bipolar I patients can experience depressive symptoms (of any severity) during a manic episode and to be willing to invite patients to complete a patient questionnaire during a consultation (although patient participation was voluntary). Psychiatrists were selected to ensure regional spread across the UK and to include a mix of hospital- and office-based psychiatrists.

For a patient to be included in the study, they were required to be an adult (≥18 years old) and currently diagnosed with bipolar I disorder, the diagnosis having been made at least 1 year previously. They were also required to have had a manic episode within approximately the past 3 months and to be currently receiving treatment for this period of mania (but not necessarily currently fulfilling full criteria for a manic episode).

#### Study design

Each psychiatrist invited five patients to complete the one-page M.I.N.I. module questionnaire (which had been finalised during Phase I). These patients did not include any who had participated in Phase I. The patient questionnaires were completed on paper on a voluntary basis and returned via post.

The psychiatrists then completed a 30-min online survey, which comprised 5 min of profiling questions followed by the completion of patient record forms for the same five patients who had been invited to complete the M.I.N.I. module. The profiling questions were used for screening purposes and to capture information regarding the psychiatrists’ caseload and setting, together with information about their practice, such as the percentage of patients seen experiencing depressive symptoms during manic episodes and the tools currently employed to assess depressive symptoms. The patient record forms captured information on each patient's demographic characteristics, the onset of their most recent manic episode, treatments received for their manic episode and the psychiatrist's diagnosis, made according to DSM-5 criteria during a clinical interview.

#### M.I.N.I. module

The M.I.N.I. module consists of nine questions that assess the presence/absence of depressive features (six symptoms), as outlined in DSM-5 (Additional file 1). The questions were based on the wording of the validated version of the M.I.N.I. (Lecrubier et al. 1997; Sheehan et al. 1998) with slight changes after Phase I. Compared with the original structured diagnostic interview version of the M.I.N.I., this self-questionnaire version was simplified using a points system for scoring. For questions split into two parts (i.e. questions 2, 4 and 5), patients were counted as having that symptom if 'yes’ was selected for at least one part. If patients answered 'yes’ to both parts of the question, it was counted as only one symptom to correspond with the DSM-5 criteria count.

#### Data analysis

The M.I.N.I. module was validated by comparing depressive symptoms reported by the patients themselves using the M.I.N.I. module with those reported by their psychiatrist assessing the presence of DSM-5 criteria during the clinical interview. The six depressive symptoms assessed were depressed mood, diminished interest/pleasure, psychomotor retardation, fatigue/loss of energy, worthless/guilty feelings and suicidal thoughts. The proportions of patients experiencing two or less or at least three depressive symptoms during a manic episode, as reported by patients and psychiatrists using the M.I.N.I. module and DSM-5 criteria, respectively, were compared. The degree of agreement between the M.I.N.I. module and DSM-5 criteria (as assessed by psychiatrists) was calculated as the sum of 'yes/yes’ , 'no/no’ and 'don't know/not answered’ responses, while the degree of disagreement was calculated as the sum of all other response combinations.

Cohen’s kappa coefficient (*κ*) was used for calculating inter-rater agreement and defined as follows:

where Pr(*a*) is the relative observed agreement between the M.I.N.I. module and psychiatrist-rated DSM-5 criteria and Pr(*e*) is the hypothetical probability of chance agreement, using the observed data to calculate the probabilities of each observer randomly saying each category. If there is complete agreement, *κ* = 1; if there is no agreement, other than what would be expected by chance (as defined by Pr(*e*)), then *κ* = 0.

The sensitivity (i.e. ability to accurately identify the presence of a symptom), specificity (i.e. ability to accurately identify the absence of a symptom), positive predictive value (PPV) and negative predictive value (NPV) were calculated for the M.I.N.I. module's ability to identify patients with at least three depressive symptoms (i.e. to identify the presence of the DSM-5 'With Mixed Features’ specifier), compared with psychiatrist-rated DSM-5 criteria during clinical interviewing. Sensitivity was calculated as the number of true positive results divided by the number of true positive results plus the number of false negative results. Specificity was calculated as the number of true negative results divided by the number of true negative results plus the number of false positive results. PPV was calculated as the number of true positive results divided by the number of true positive results plus the number of false positive results. NPV was calculated as the number of true negative results divided by the number of true negative results plus the number of false negative results. A true positive result was recorded when the M.I.N.I. module and the DSM-5 (as assessed by the psychiatrist) both detected the presence of a symptom ('yes/yes’). A true negative result was recorded when the M.I.N.I. module and the DSM-5 both detected the absence of a symptom ('no/no’). A false positive result was recorded when the M.I.N.I. module detected the presence of a symptom while the DSM-5 detected its absence ('yes/no’). A false negative result was recorded when the M.I.N.I. module detected the absence of a symptom while the DSM-5 detected its presence ('no/yes’).

*κ*, sensitivity, specificity, PPV and NPV were also calculated for the M.I.N.I. module's ability to detect each of the six individual depressive symptoms assessed.

## Results

### Phase I

Overall, 12 of 20 (60%) patients participating in Phase I presented with mixed features (at least three depressive symptoms). Feedback from all patients was analysed and the wording of the M.I.N.I. module was revised and finalised, as shown in Additional file 1. For example, in the introductory sentence, 'nearly every day’ was changed to 'almost every day’ at the suggestion of some patients, since several understood 'nearly every day’ to mean 'most days’. For questions where different symptoms were assessed together (questions 2, 4 and 5), several patients found it difficult to provide a 'yes’ or 'no’ response, so these questions were split into two parts ('a’ and 'b’) in the finalised version.

### Phase II

#### Psychiatrist and patient characteristics

A total of 23 psychiatrists were included in the study and altogether they completed patient record forms for a total of 115 patients (i.e. five patients each). Of these 115 patients, 99 (86.1%) completed and returned the M.I.N.I. module questionnaire. Details of the psychiatrists' practice characteristics are shown in Table 1. Psychiatrists were recruited from across the UK and had a mean duration of psychiatric experience of 17.8 years. Their mean caseload was 139 patients/month, which included a mean of 47 patients with bipolar I disorder per month, 52.3% of whom were experiencing a manic episode at any one time. Patient characteristics are summarised in Table 1. The mean age of patients was 43.7 years and 53.9% were female. All patients had recently experienced a manic episode (as per inclusion criteria), and all consultations took place during November 2012, in accordance with the prospective design of the study.

**Table 1:** **Summary of psychiatrist practice details and patient characteristics (Phase II)**

Characteristics	Value
(A) Psychiatrists	*n* = 23
Years qualified, % (*n*)	
5 to 9	4.3 (1)
10 to 14	39.1 (9)
15 to 19	8.7 (2)
≥20	47.8 (11)
Mean years qualified, *n*	17.8
Patient caseload	
Mean total number of patients/month, *n*	139
Mean number of patients with bipolar I disorder/month, *n*	47
Patients experiencing a manic episode at any one time, %	52.3
Country regions, % (*n*)	
London and South England	43.5 (10)
North England and Midlands	43.5 (10)
Scotland, Wales and Northern Ireland	13.0 (3)
Patient setting, % (*n*)^a^	
Inpatients	17.4 (4)
Outpatients	52.2 (12)
Both	30.4 (7)
(B) Patients	*n* = 155
Sex, % (*n*)	
Male	46.1 (53)
Female	53.9 (62)
Age range (years), % (*n*)	
18 to 25	15.7 (18)
26 to 35	20.9 (24)
36 to 45	24.3 (28)
46 to 55	18.3 (21)
56 to 66	13.0 (15)
>66	7.8 (9)
Mean age, years	43.7
Onset of most recent/current manic episode, % (*n*)	
Current month	18.3 (21)
1 month ago	33.0 (38)
2 months ago	28.7 (33)
3 months ago	17.4 (20)
4 months ago	2.6 (3)
Date of consultation, % (*n*)	
1 to 18 November 2012	55.7 (64)
19 to 25 November 2012	20.9 (24)
26 to 30 November 2012	23.5 (27)

^a^Mean percentage of psychiatrists spending time in each setting.

#### Psychiatrist profiling

**Figure 1:**
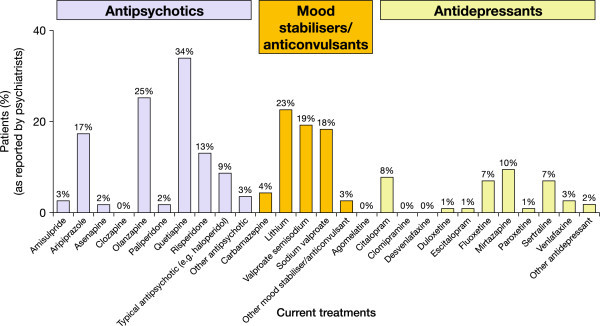
**Treatments received by patients to manage their manic episodes, as reported by psychiatrists (Phase II).**

When psychiatrists were asked what proportion of their patients with bipolar disorder exhibit depressive symptoms (regardless of severity) during a manic episode, 78% reported values of 21% to 50%; the mean proportion reported was 36%. When asked how they currently assess depressive symptoms in patients with bipolar disorder, all the psychiatrists replied that they do so through clinical examination and questioning of the patient, and most (96%) replied that they also speak to the patient's caregivers; 65% use diagnostic criteria (e.g. DSM-IV, ICD-10), and 22% use a validated tool to measure depression. Psychiatrists reported that patients receive a variety of treatments to manage their manic episodes; most commonly, antipsychotics (quetiapine, 34%; olanzapine, 25%) and mood stabilisers (lithium, 23%; valproate semisodium, 19%; sodium valproate, 18%), with approximately 10% of patients also prescribed antidepressants (Figure 1).

#### Validation of M.I.N.I. module against psychiatrist-rated DSM-5 criteria

**Figure 2:**
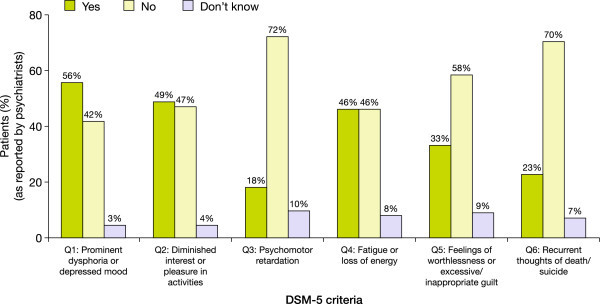
**Depressive symptoms experienced by patients during a manic episode, as evaluated by psychiatrists (Phase II).**

**Figure 3:**
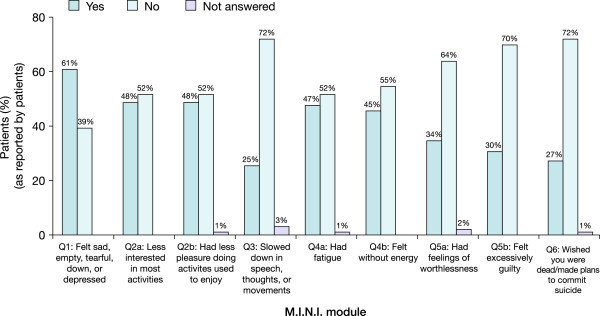
**Depressive symptoms experienced by patients during a manic episode, as reported by patients (Phase II).**

**Figure 4:**
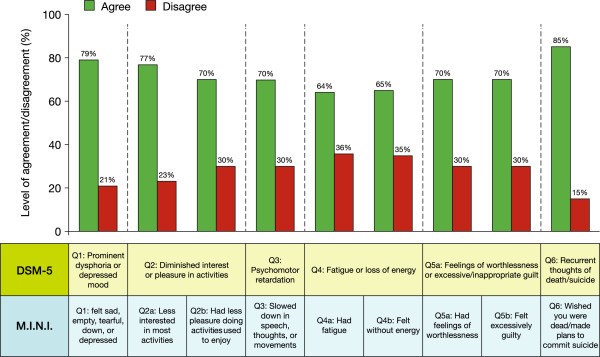
**Level of agreement/disagreement between depressive symptoms reported by psychiatrists (DSM-5) and patients (M.I.N.I. module).**

Psychiatrists diagnosed their patients during a clinical interview using DSM-5 criteria and logged the results on each patient's record form. Overall, psychiatrists reported that 74.8% (86/115) of patients experienced at least one depressive symptom during a manic episode. The most frequently observed depressive symptoms reported by psychiatrists were depressed mood, diminished interest/pleasure and fatigue; more severe symptoms (e.g. psychomotor retardation, recurrent thoughts of death/suicide) were less frequently observed (Figure 2). Similarly, 81.8% (81/99) of patients completing the M.I.N.I. module reported experiencing at least one depressive symptom during a manic episode. Consistent with the psychiatrists' DSM-5 diagnoses, patients mainly reported experiencing depressed mood, diminished interest/pleasure and fatigue; more severe symptoms again were less frequently reported (Figure 3). When matched records were compared, there was a high level of agreement in symptom reporting between psychiatrists and patients across all symptoms; the highest levels of agreement were for the most common symptom (depressed mood, 79%) and the most severe symptom (suicidal thoughts, 85%), and the lowest level of agreement was for the least severe symptom (fatigue, 64% to 65%) (Figure 4).

**Figure 5:**
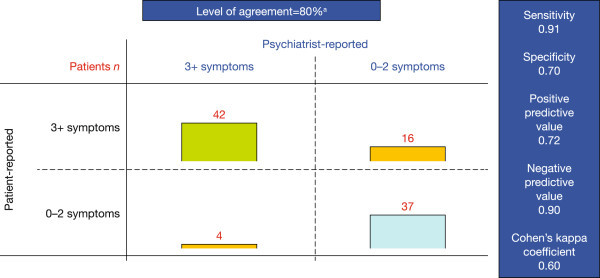
**Validation of the M.I.N.I. module for assessment of the DSM-5 'With Mixed Features’ specifier.** Number of patients experiencing less than two or at least three depressive symptoms during a manic episode, as reported by patients using the M.I.N.I. module versus psychiatrists using DSM-5 criteria during clinical interviewing. For questions that were split into two parts for patients (M.I.N.I. module questions 2, 4 and 5), patients were counted as having that symptom if 'yes’ was selected for at least one part. If patients answered 'yes’ to both parts of the question, it was counted as only one symptom to correspond with the DSM-5 criteria count^a^.

Overall, there was 80% agreement between the M.I.N.I. module and psychiatrist-rated DSM-5 criteria regarding the number of depressive symptoms experienced by bipolar patients during a manic episode (Figure 5). According to psychiatrists, 46.5% (46/99) of patients experienced at least three depressive symptoms during a manic episode and therefore met DSM-5 criteria for the 'With Mixed Features’ specifier. Patients were more likely than psychiatrists to report the presence of at least three depressive symptoms, with 58.6% (58/99) of patients reporting at least three depressive symptoms using the M.I.N.I. module.

The overall sensitivity and specificity of the M.I.N.I. module in identifying patients meeting the criteria for the DSM-5 specifier were 0.91 and 0.70, respectively; the overall PPV, NPV and *κ* were 0.72, 0.90 and 0.60, respectively (Figure 5). The sensitivity of the M.I.N.I. module in identifying individual depressive symptoms ranged from 0.63 for psychomotor retardation to 0.90 for suicidal thoughts, and its specificity ranged from 0.63 for diminished interest/pleasure to 0.90 for suicidal thoughts (Table 2). PPVs ranged from 0.50 for psychomotor retardation to 0.79 for depressed mood, NPVs ranged from 0.80 for fatigue/loss of energy to 0.97 for suicidal thoughts and *κ* ranged from 0.42 for psychomotor retardation to 0.74 for suicidal thoughts (Table 2).

**Table 2:** **Validation of the M.I.N.I. module to identify individual depressive symptoms (Phase II)**

Symptom	Sensitivity	Specificity	PPV	NPV	***κ***
Depressed mood	0.87	0.73	0.79	0.82	0.60
Diminished interest/pleasure	0.87	0.63	0.69	0.84	0.50
Psychomotor retardation	0.63	0.82	0.50	0.89	0.42
Fatigue/loss of energy	0.81	0.69	0.70	0.80	0.50
Worthless/guilty feelings	0.76	0.77	0.66	0.85	0.51
Suicidal thoughts	0.90	0.90	0.72	0.97	0.74

Proportions of patients experiencing each depressive symptom during a manic episode, as reported by patients using the M.I.N.I. module compared with those reported by psychiatrists using DSM-5 criteria during clinical interviewing. *κ*, Cohen’s kappa coefficient; NPV, negative predictive value; PPV, positive predictive value.

## Discussion

This prospective, real-world study found that the M.I.N.I. module demonstrates good concurrent validity with psychiatrists' evaluation of DSM-5 mixed features in patients with bipolar mania. The M.I.N.I. module showed an excellent capacity to detect mixed features with a limited risk of over-diagnosis. Although the psychiatrists participating in the study estimated that approximately one third (36%) of bipolar I patients experience depressive symptoms within a manic episode, this figure doubled when they were presented with the DSM-5 criteria for mixed features, with 75% of patients experiencing at least one depressive symptom. Indeed, according to the psychiatrists, 46% of patients met the DSM-5 criteria for the 'With Mixed Features’ specifier. The agreement between the psychiatrists' assessment and the patients' assessment using the M.I.N.I. module to detect the presence of the DSM-5 specifier was substantial, with a *κ* of 0.60. The M.I.N.I. module's sensitivity and specificity were also high (0.91 and 0.70, respectively), and there was substantial agreement between psychiatrists and patients with regard to the presence/absence of specific depressive symptoms. Taken together, these findings demonstrate that the M.I.N.I. module used as a self-questionnaire is a robust assessment tool for identifying hypomanic and manic bipolar patients meeting the criteria for the DSM-5 'With Mixed Features’ specifier. This version could be used as an optional module added to a clinical interview. Indeed, a 'classical’ clinician module could be developed and added to the M.I.N.I. in order to improve the overall concordance with psychiatrists' diagnosis. It is important to highlight that the M.I.N.I. module has been designed specifically to evaluate the DSM-5 specifier for (hypo-)manic episodes in patients with bipolar I or II disorders; therefore, a corresponding module for evaluating 'hypomanic features’ during bipolar or unipolar major depressive episodes should be developed. It should also be pointed out that the M.I.N.I. module is currently not applicable to paediatric bipolar disorder.

A potential limitation of the M.I.N.I. module is that the item corresponding to psychomotor retardation in the DSM-5 'With Mixed Features’ specifier (Q3 of the M.I.N.I. module) simply asks the patient 'Since you have been experiencing your current manic episode, have you almost every day had times when you were slowed down in your speech, thoughts, or movements?’ , whereas DSM-5 states that psychomotor retardation should be 'observable by others; not merely subjective feelings of being slowed down’ (American Psychiatric Association 2013b). Indeed, validation of the M.I.N.I. module showed that Q3 had the lowest sensitivity and *κ* values of the six items; however, it had one of the highest specificity values (0.82), thereby limiting the likelihood of a false positive result and, hence, over-diagnosis (Table 2). In addition, psychomotor retardation was found to be the depressive symptom that patients presented with least frequently, according to physicians using the DSM-5 'With Mixed Features’ specifier during clinical interviewing (Figure 2).

The replacement of the DSM-IV-TR diagnostic criteria for a mixed episode with the new 'With Mixed Features’ specifier in DSM-5 demonstrates a growing recognition of the clinical relevance of subthreshold depression during mania. Patients experiencing depressive symptoms during a manic episode represent a more severe population. Outcomes and prognosis in patients with depressive symptoms during a manic episode are poorer than those in patients with pure mania, with higher recurrence rates, a higher prevalence of physical co-morbidity, a higher risk of hospitalisation, more frequent co-morbid substance abuse, and a greater risk of suicidal ideation and suicide attempts (González-Pinto et al. 2007, 2011; Goldberg and McElroy 2007; Valentí et al. 2011). Moreover, depressive symptoms during mania are highly prevalent, occurring in approximately one third of patients with bipolar disorder (González-Pinto et al. 2007, 2011). Depending on the predominant symptoms, patients with mixed features may demonstrate a poor response to lithium and/or become less stable when taking antidepressants (American Psychiatric Association 2013a), therefore requiring treatment that is tailored to their particular needs. The DSM-IV-TR criterion that patients must simultaneously meet all criteria for an episode of mania and an episode of major depression in order to be diagnosed with a mixed episode have meant that the needs of many individuals in this severe patient subgroup have so far not been adequately met. It is hoped that the DSM-5 'With Mixed Features’ specifier will address this unmet need. Although medications that are effective in treating mixed episodes as defined by DSM-IV-TR criteria may also be effective in treating mixed features according to criteria for the DSM-5 specifier, this needs to be confirmed in further studies (Vieta and Valentí 2013).

## Conclusions

The results of this study indicate that the M.I.N.I. module administered as a self-questionnaire is a robust assessment tool for assessing depressive symptoms in bipolar patients with hypomania or mania, according to the DSM-5 'With Mixed Features’ specifier. Due to its simplicity, the M.I.N.I. module can easily be incorporated into the routine psychiatric evaluation of patients with manic episodes. It is also a valuable standardised tool for clinical and epidemiological research in bipolar disorder.

## Authors’ information

Both TH and EW were involved in the development and validation of the original M.I.N.I. (Lecrubier et al. 1997; Sheehan et al. 1998). The M.I.N.I. module can be obtained from the authors at adep.paris@noos.fr.

## Electronic supplementary material

Additional file 1: **M.I.N.I. (hypo-)manic episode 'With Mixed Features’ module: patient version.** (PDF 2 MB)

## Abbreviations

DSM-5: Diagnostic and Statistical Manual of Mental Disorders, fifth edition
DSM-IV-TR: Diagnostic and Statistical Manual of Mental Disorders, fourth edition-text revision
ICD-10: International Statistical Classification of Diseases and Related Health Problems tenth revision
κ: Cohen’s kappa coefficient
M.I.N.I.: Mini International Neuropsychiatric Interview
NPV: Negative predictive value
PPV: Positive predictive value
Pr(a): Relative observed agreement
Pr(e): Hypothetical probability of chance agreement.
