# Supplementary material for: Evaluating depressive symptoms in hypomanic and manic episodes using a structured diagnostic tool: validation of a new Mini International Neuropsychiatric Interview (M.I.N.I.) module for the DSM-5 'With Mixed Features’ specifier
Source: Int J Bipolar Disord. 2013 Oct 17;1:21. doi: 10.1186/2194-7511-1-21 (PMC4230688; doi:10.1186/2194-7511-1-21)
Supplement: Supplementary file 1 — Additional file 1: M.I.N.I. (hypo-)manic episode 'With Mixed Features’ module: patient version. (PDF 2 MB) [file 40345_2013_21_MOESM1_ESM.pdf]

M.I.N.I.

Mini International Neuropsychiatric Interview

(Hypo-) Manic Episode “with Mixed Features” - DSM-5 module

PATIENT VERSION

The Mini International Neuropsychiatric Interview, better known as the M.I.N.I., is a Structured Diagnostic Interview. It was developed during a close Franco-American partnership that began in 1992 to assist clinicians to conduct psychiatric diagnoses most often encountered, by using the criteria of international classifications (ICD-10 and DSM-IV). It has now become a benchmark in both research and clinical practice. It has been translated into 65 languages. The original M.I.N.I. contains 17 modules.

The present module was developed to evaluate the DSM-5 specifier “with Mixed features” for (hypo-)manic episodes.
This version can be filled in by patients.

Rating
For this patient version, a point system was introduced based on one point for each YES answer selected. For questions n° 2, 4 and 5, one point is given if the patient answers Yes to either a or b, or both.

Diagnostic algorithm
If the total number of points is equal to or greater than 3, the patient presents a *probable* (Hypo-) Manic Episode *with Mixed Features*.
To confirm the diagnosis, ensure that the mixed symptoms are observable by others and represent a change from the person’s usual behavior and that the mixed symptoms are not due to the direct physiological effects of a substance (e.g., a drug of abuse, a medication, or other treatment).

Note: The M.I.N.I. is a tool to **help** diagnosis but cannot replace a thorough clinical examination.

Y. Lecrubier, E. Weiller, T. Hergueta, P. Amorim, L.I. Bonora, J.P. Lépine (Hôpital de la Salpêtrière -PARIS, FRANCE) D. Sheehan, J. Janavs, R. Baker, K.H. Sheehan, E. Knapp, M. Sheehan (University of South Florida-TAMPA, USA)

© 2012 T. Hergueta. To obtain the original version, send an email to *adep.paris@noos.fr*

The below questions refer to symptoms people might experience either simultaneously or fluctuating with manic symptoms, during a manic episode.

Please read each question carefully.

If the question describes symptoms you have experienced almost every day since the beginning of your current manic episode, circle YES.

If the question does not describe how you have been feeling, or if you are in doubt, circle NO.

(*Ignore the grey areas*)

		Since you have been experiencing your current manic episode, have you almost every day had times when:			POINTS *Do not fill in*
1	▪	You felt sad, empty, tearful, down, or depressed?	NO	YES	⊠ 1
2a	▪	You were less interested in most activities?	NO	YES	
2b	▪	You had less pleasure doing the activities you used to enjoy?	NO	YES	⊠ 1
3	▪	You were slowed down in your speech, thoughts, or movements?	NO	YES	⊠ 1
4a	▪	You had fatigue?	NO	YES	
4b	▪	You felt without energy?	NO	YES	⊠ 1
5a	▪	You had feelings of worthlessness?	NO	YES	
5b	▪	You felt excessively guilty?	NO	YES	⊠ 1
6	▪	You wished you were dead, considered hurting yourself, made plans to commit suicide or attempted suicide?	NO	YES	⊠ 1
				TOTAL =	

IF THE **TOTAL** NUMBER OF POINTS IS **EQUAL TO OR GREATER THAN 3**, THE PATIENT PRESENTS A PROBABLE **(HYPO-) MANIC EPISODE *WITH MIXED FEATURES***

Y. Lecrubier, E. Weiller, T. Hergueta, P. Amorim, L.I. Bonora, J.P. Lépine (Hôpital de la Salpêtrière -PARIS, FRANCE) D. Sheehan, J. Janavs, R. Baker, K.H. Sheehan, E. Knapp, M. Sheehan (University of South Florida-TAMPA, USA)

© 2012 T. Hergueta. To obtain the original version, send an email to *adep.paris@noos.fr*
